# Primary and postoperative radiotherapy in acute neurological symptoms due to malignant spinal compression: retrospective analysis from a German university hospital

**DOI:** 10.1186/s12885-025-14106-y

**Published:** 2025-04-23

**Authors:** Manuel Guhlich, Teresa Esther Maag, Markus Anton Schirmer, Tatiana Andrea Chacón Quesada, Dorothee Mielke, Stefan Rieken, Martin Leu, Leif Hendrik Dröge

**Affiliations:** 1 Clinic of Radiotherapy and Radiation Oncology, University Medical Center Göttingen, Robert-Koch-Str. 40, 37075 Göttingen, Germany; 2 Department of Neurological Surgery, University Medical Center Göttingen, Robert-Koch-Str. 40, 37075 Göttingen, Germany; 3 Department of Neurosurgery, University Medical Center Augsburg, Stenglinstr. 2, 86156 Augsburg, Germany; 4 Quality Conferences Office at the Clinical State Registry Baden-Württemberg GmbH, Baden-Württemberg Cancer Registry (BWCR), Stuttgart, Germany

**Keywords:** Spinal cord compression, Metastatic spinal cord compression, SCC, MSCC, Radiotherapy, Radiation therapy, Bone metastasis, Acute neurology, Emergency radiation therapy, Retrospective analysis

## Abstract

Malignant spinal cord compression (MSCC) can lead to immediate neurological impairment. In order to preserve and, optimally, restore neurological functions, urgent treatment (usually, within 24 h) is necessary. Treatment options mainly consist of decompressive surgery (DS) and / or radiotherapy (RT) combined with steroids. Whereas historically, RT was the treatment of choice, DS has become standard of care, where applicable. Despite a variety of excellent studies, real world data of treatment in a large academic center is currently underrepresented. We performed a retrospective analysis of patients treated for MSCC in our department of radiotherapy between 1998 and 2018 (*n* = 131), evaluating treatment, achievement of clinically determined improvement of neurological functions as well as overall survival (OS) and treatment-related toxicity. Kaplan-Meier estimator was used for survival statistics, log rank test for survival time comparisons, univariable and multivariable Cox regression and logistic regression for assessing potential impacts of variables on survival and symptom relief. 42.7% of patients had DS before RT (*n* = 56), 57.3% (*n* = 75) received RT without DS. Symptom relief was achieved in 41.2% of all patients (*n* = 54, *n* = 26 of those had DS before RT, *p* = 0.12). RT completed as intended (*p* < 0.001) was statistically significant for symptom relief, wherein symptom relief (*p* < 0.001), completion of RT course as intended (*p* = 0.01) and more recent treatment dates (*p* = 0.002) were independent predictors for OS. We herein present a large cohort of patients treated for MSCC in our academic center, representing real world treatment data currently lacking in literature.

**Supplementary Information:**

The online version contains supplementary material available at 10.1186/s12885-025-14106-y.

## Background

Spinal cord compression (SCC), predominantly caused by metastatic disease, frequently occurs in patients suffering from advanced cancers of various types and is an oncological emergency [1]. Immediate diagnostic and therapeutic workup is essential for preserving remaining as well as potentially regaining lost neuronal function [2]. In addition to potential neuronal impairment, patients frequently endure severe pain [1, 3] and unstable vertebrae [4]. Historically, radiotherapy (RT) and corticosteroids were treatments of choice [5, 6]. Nowadays, in most cases, primary decompressive surgery (DS) is performed, followed by consolidative RT [7, 8]. This treatment strategy is mainly based on a prospective randomized trial, reporting significantly better outcomes when performing DS upfront RT [8]. However, the study design excluded certain primary tumors, which have shown to be highly sensitive to RT, e.g. myeloma and lymphoma. Furthermore, patients had to present in a good performance score to be cleared for surgery as well as a life expectancy of at least three months. This resulted in a highly selected patient cohort, not fully representing clinical reality. Subsequently, only a few retrospective studies compared DS preceding RT to RT alone. In 2010, a matched pair analysis reported similar post-treatment outcomes in neuronal functions [9]. In 2011, a comparative study reported an apparent benefit in terms of improved functional outcome for patients receiving DS (but not laminectomy) prior to RT specifically for unfavorable primary tumors [10]. A recently published matched-pair study including data from prospectively evaluated patients supports these findings. The study reports higher rates of motor function improvement in the surgery upfront RT-cohort [11]. Considering peri- and intraoperative risks, treatment decisions as well as supportive therapies should be made in an experienced interdisciplinary team, involving surgeons, radiation oncologists and palliative care physicians [12]. Following this approach, we performed the present retrospective study in order to broaden the current literature. The aim was to evaluate and compare treatment outcomes in patients receiving RT with or without upfront DS when presenting with symptomatic SCC.

## Methods

This single center study retrospectively analyzed patients receiving emergency RT with or without DS for SCC. RT took place at the Department of Radiotherapy and Radiooncology at the University Medical Center in Göttingen, Germany, between 01/1998 and 12/2018. Patients and their respective diagnoses were identified by systematic keyword screening for “paraplegia”. Data were extracted from patient records and RT treatment planning system (Varian Eclipse, version 15.6, Varian Medical Systems, Palo Alto, USA). Patient follow-up was assessed by reviewing hospital internal data processing systems (ixserv.4, version R20.3, ix.mid software technology, Köln, Germany and ONKOSTAR, version 2.9.8, IT-Choice Software AG, Karlsruhe, Germany). The study was conducted according to the guidelines of the Declaration of Helsinki and approved by the Ethics Committee of the University Medical Center Göttingen (protocol code 19/5/21, date of approval: 07th June 2021).

Primary endpoint was achievement of symptom relief in terms of any clinically determined improvement of neurological functions. Due to insufficient data, this could not be analyzed in detail. Secondary endpoints were overall survival and treatment-related toxicity according to CTCAE V5.0 [13].

Statistical analysis was conducted using SPSS (v. 27) and R (v. 4.0.2) with the “KMWin” (Kaplan–Meier for Windows) plugin [14]. For survival statistics, we used the Kaplan-Meier estimator. Survival time comparisons were performed by log-rank tests. Variables used in the analysis were selected retrospectively based on data patterns. Univariable cox regression was used to assess the impact of variables on survival, while univariable logistic regression was applied similarly for symptom relief. We considered *p*-values < 0.05 as statistically significant. Prior to conducting multivariable analyses high pairwise inter-correlation (i.e. *p* < 0.001 according to Kendall’s tau b analysis) was tested and if this was the case, the corresponding inter-correlated variable was removed from the multiple statistical models.

## Results

### Patients

**Fig. 1:**
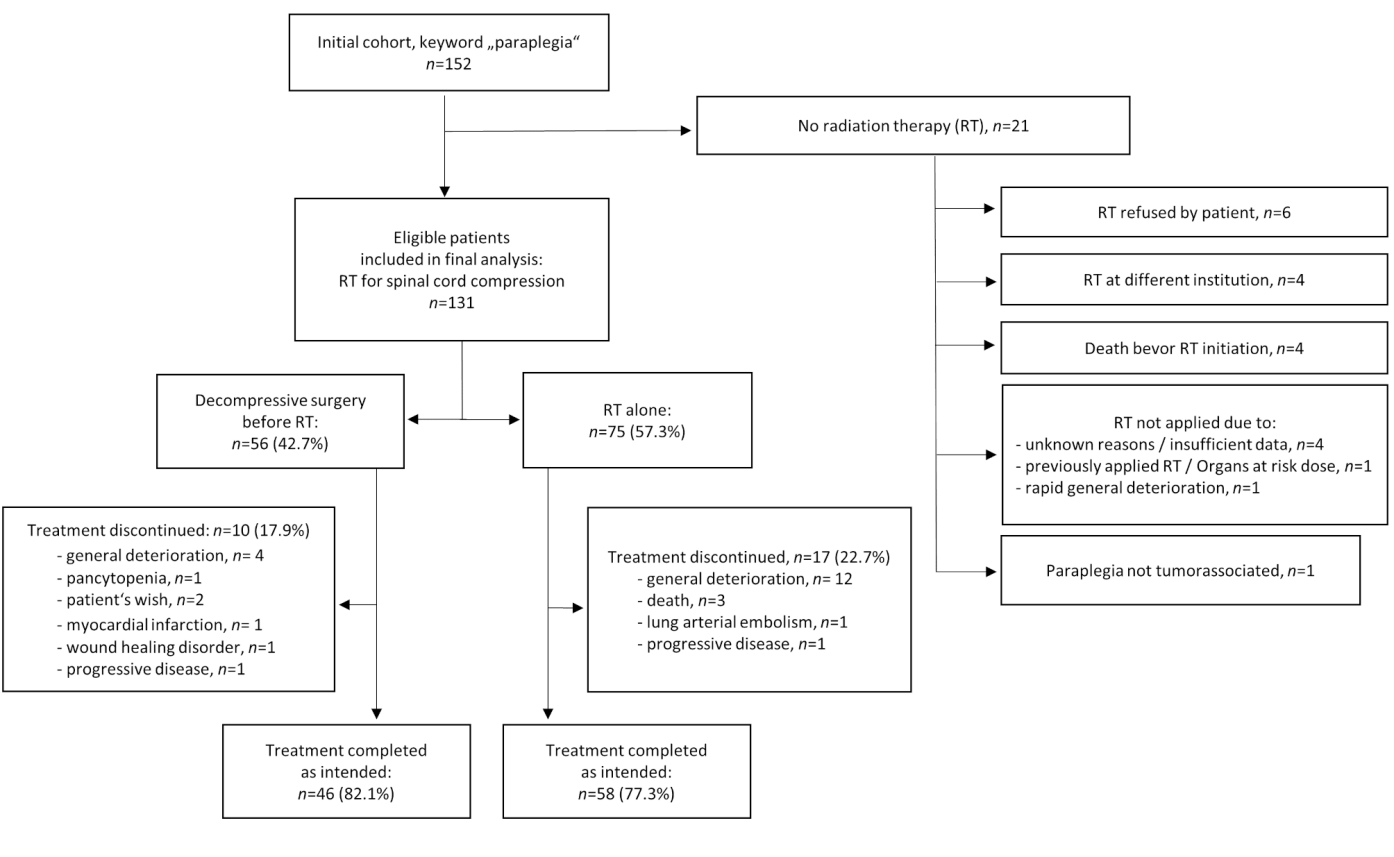
Consort flowchart

A total of 131 patients were eligible for analysis. Please refer to Fig. 1 for a CONSORT flowchart. Mean age at initial diagnosis was 59 years (range, 30–89), 59.5% of patients were male (*n* = 78), 40.5% female (*n* = 53). Mean Charlson Comorbidity Index (CCI) was 5.7. DS before RT was performed in 56 patients (42.7%). 14 patients (10.7%) were diagnosed with their respective tumor diagnoses at immediate presentation with SCC. Please refer to Table 1 for patient, disease and general treatment characteristics.

**Table 1:** Patient, disease and treatment characteristics

Patients, *N*	131
Age (years), median (min–max)	59 (30–89)
Sex: female: male, N (%)	53 (40.5): 78 (59.5)
**Charlson Comorbidity Index**,** N (%)**	
1–3	18 (13.7)
4–6	96 (73.3)
7–10	19 (14.5)
**Disease and Treatment characteristics**,** N (%)**	
SCC as first symptom of disease	14 (10.7)
DS before RT	56 (42.7)
Chemotherapy, prior to SCC	70 (53.4)
Immunotherapy, prior to SCC	15 (11.5)

DS = decompressive surgery, RT = Radiotherapy, SCC = spinal cord compression

Main primary tumor sites were breast and prostate cancer, accounting for 51 patients (38.9%) in total. Please refer to Table 2 for specifics concerning primary tumor sites. This aligns to 22, 73, and 22 cases with low, intermediate, and high radiosensitivity, respectively.

**Table 2:** Primary tumor entities causing SCC sorted by frequency in our study cohort. Radiosensitivity was grouped in three categories: high, intermediate, and low. N.a.=not applicable

Tumor entity	*N* (%)	Radiosensitivity
Breast Carcinoma	27 (20.6)	Intermediate
Prostate Carcinoma	24 (18.3)	Intermediate
Cancer of unknown primary (CUP), not identified by histology or multiple maligancies	14 (10.7)	n.a.
Non-small cell lung carcinoma	11 (8.4)	Intermediate
Plasmocytoma / Multiple Myeloma	10 (7.6)	High
Renal Cell Carcinoma	9 (6.9)	Low
Small cell lung carcinoma	8 (6.1)	High
Rectum Carcinoma	6 (4.6)	Intermediate
Melanoma	3 (2.3)	Low
Non-Hodgkin lymphoma	3 (2.3)	High
Colon Carcinoma	2 (1.5)	Intermediate
Hepatocellular Carcinoma	2 (1.5)	Low
Malignant fibrous histiocytoma	1 (0.7)	Low
Langerhans cell histiocytosis	1 (0.7)	Intermediate
Bladder Carcinoma	1 (0.7)	Low
Glioblastoma	1 (0.7)	Low
Adenocarcinoma of the tear gland	1 (0.7)	Intermediate
Esophageal Carcinoma	1 (0.7)	Intermediate
Undifferentiated Sarcoma	1 (0.7)	Low
Neurofibroma	1 (0.7)	Low
Adrenal Carcinoma	1 (0.7)	Low
Pancreatic Carcinoma	1 (0.7)	Low
Nasopharynx Carcinoma	1 (0.7)	High
Cholangiocellular Carcinoma	1 (0.7)	Low

RT was applied as intended in 79.4% of patients (*n* = 104) and completed early in 20.6% (*n* = 27), mainly due to deterioration of general condition or patients’ choice. Treatment was mainly hypofractionated. Stereotactic body radiotherapy and/or simultan integrated boosts were not applied as they were not established in the clinic at that time. Intended RT dose was according to the decision of the attending physician. Generally, this consisted of an intended dose of 30 Gy (10fractions/3Gy), as was the usual prescription of the department in this specific clinical situation. It was prescribed and delivered dose/fractionation for 80 patients (61% of all eligible patients). Additional 16 patients (12.2%) were prescribed 10*3Gy, but did not receive the intented dose due to various reasons. Seven patients received 13*3 Gy, four patients 20*2 Gy. Only two patients had an intended dose above 40 Gy: one patient suffering from spinal cord compression by a dedifferentiated adenocarcinoma of the lung, one suffering from neurofibroma; each being described 28*1.8 Gy. Further deviations from these dose concepts were due to preirraditions (*n* = 8; 6.1%) Please refer to Fig. 1 for details concerning preliminary RT abortion and to Table 3 for RT treatment details, including acute treatment-related side effects.

**Table 3:** Radiotherapy treatment, symptom relief and acute treatment-related side effects (according to CTCAE V5.0 [13])

**Course of Radiotherapy (RT)**: ***N*** (**%**)	
Intended RT complete	102 (77.9)
Intended RT incomplete	29 (22.1)
**RT dose and technique: N (%)**	
Dose, median (min–max)	30.0 Gy (2.0–50.4)
3D conformal RT (3DcRT)	129 (98.5)
Volumetric modulated Arc Therapy (VMAT)	2 (1.5)
Acute treatment-related side effects (CTCAE V5.0): N (%)*	
Skin erythema, Grade 1	5 (3.8)
Esophagitis, Grade 1	9 (6.9)
Emesis, Grade 1	5 (3.8)
Emesis, Grade 2	1 (0.7)
Enteritis, Grade 1	3 (2.3)
Enteritis, Grade 2	1 (0.7)
Proctitis, Grade 1	1 (0.7)
Acute side effects, any	16 (12)

RT = Radiotherapy, DS = decompressing surgery. *Acute treatment related side effects were scored due to the current CTCAE version (1.0 up to 5.0, depending on treatment date) and rescored to the current version 5.0. There were no toxicities exceeding grade 2

### Symptom relief and treatment compliance

For a total of 54 patients (41.2%), symptom relief in terms of a clinically determined improvement of neurological functions was achieved. Unfortunately, data was insufficient to account for detailed analysis, as stated in the methods section. The documented improvement mainly accounted for a regain in motor neuron function after DS or at the end of the RT course. Within those who improved, 26 (48.1%) had DS (decompression and/or stabilization) ahead of RT, 28 patients received RT alone. In 96.3% of patients who achieved a symptom relief, RT was completed as initially intended. Please refer to Table 4 for details on symptom relief and RT course completion.

**Table 4:** Symptom relief and RT course completion

	Symptom relief: *N* (%)	Intended RT complete: *N*(%)
**All patients** (*n* = 131)	**54** / 131 (41.2)	**104** / 131 (79.4)
DS before RT (*n* = 54)	26 / **54** (48.1)*	46 / **104** (44.2)#
RT alone (*n* = 77)	28 / **54** (51.9)*	58 / **104** (55.8)#
Patients with intended RT complete	52 / **54** (96.3)*	

RT = Radiotherapy, DS = decompressing surgery, *subgroup analysis of patients that did achieve symptom relief, #subgroup analysis of patients that did not complete the intended RT course

We tested several potential confounders concerning the outcome “symptom relief”. In a multivariable logistic regression model, applied RT dose remained statistically significant for symptom relief. Please refer to Table 5 for univariable and multivariable calculations. Univariable analysis revealed strongest associations for symptom relief for “RT completed as intended” and for “BED”; “BED” and “RT completed without interruption of ≥ 3 days” showed high correlations with “RT completed as intended” (pairwise *p* < 0.001 in Kendall’s tau b test) and thus were excluded from the multivariable logistic regression. Other than these variables did not associate with symptom relief, for instance, this was also true for the long time interval of patient recruitment and treatment over 21 years.

“RT completed as intended” exhibited Kendall’s tau b correlation coefficients of 0.72 and 0.55 with “RT completed without interruption of ≥ 3 days” and “BED”, respectively, and thus were excluded from the multivariable logistic and Cox regression models. No further multicollinearity was identified by means of linear regression as defined above in the Methodology section. Comparing parameter estimates between univariable and multivariable logistic regression revealed noticeably similar hazard ratios whereby the strong effect of “RT completed as intended” was retained in the multivariable model.

**Table 5:** Logistic regression concerning potential confounders for symptom relief

Variable	Symptom relief			
	Univariable		Multivariable	
	Odds ratio (95%-CI)	*P*-value	Odds ratio (95%-CI)	*P*-value
Age [per year]	0.98 (0.95–1.01)	0.15	0.98 (0.94–1.02)	0.27
Sex female (52) vs. male (79)	1.08 (0.53–2.19)	0.84	1.12 (0.47–2.68)	0.79
CCI > 6 (19) vs. ≤ 6 (112)	0.46 (0.16–1.36)	0.16	0.55 (0.16–1.95)	0.36
SCC as first sign of disease yes (14) vs. no (117)	1.49 (0.49–4.52)	0.48	1.49 (0.36–6.18)	0.58
Relapse of tumor yes (38) vs. no (93)	1.05 (0.49–2.26)	0.90	1.30 (0.49–3.44)	0.60
Spinal metastasis of solid tumor yes (112) vs. no (19)	0.75 (0.28–1.98)	0.56	0.41 (0.12–1.38)	0.15
Systemic therapy^§^ yes (55) vs. no (71)	1.38 (0.68–2.81)	0.38	1.05 (0.44–2.51)	0.90
Surgery conducted prior to radiotherapy, yes (56) vs. no (75)	1.66 (0.82–3.35)	0.16	1.50 (0.62–3.60)	0.37
RT completed as intended yes (103) vs. no (28)	13.26 (2.99–58.77)	**0.001**	21.56 (3.95-117.68)	4*10 ^**− 4**^
RT completed without interruption of ≥ 3 consecutive days, yes (86) vs. no (45)^%^	2.63 (1.20–5.75)	**0.02**		
Time interval between first (01/98) and last (11/18) patient irradiated [per year]	1.02 (0.96–1.09)	0.50	1.04 (0.95–1.13)	0.40
BED [per Gy]^%^	1.10 (1.04–1.16)	**< 0.001**		

^§^Any kind of systemic therapy within 12 months to radiotherapy of spinal manifestations due to SCC. In five cases, date of systemic therapy was not available and thus were omitted from analysis here. ^%^These two parameters were highly correlated with “RT completed as intended” and were thus not considered for the multivariable linear regression model. CCI = Charlson comorbidity index. SCC = spinal cord compression. RT = radiotherapy. BED = Biologically effective dose of radiotherapy

### Overall survival

**Fig. 2:**
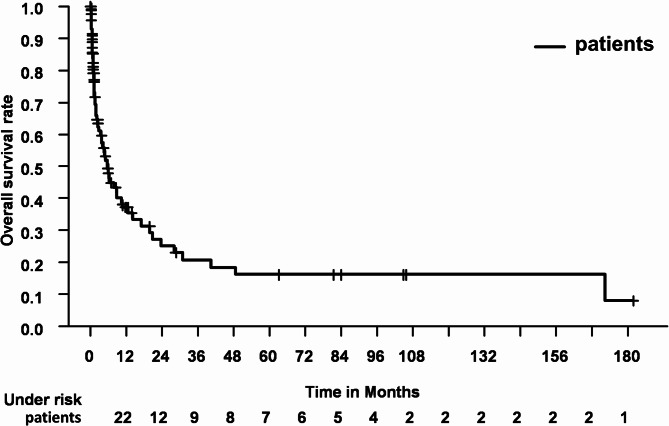
Kaplan-Meier estimate of OS for all patients

**Fig. 3:**
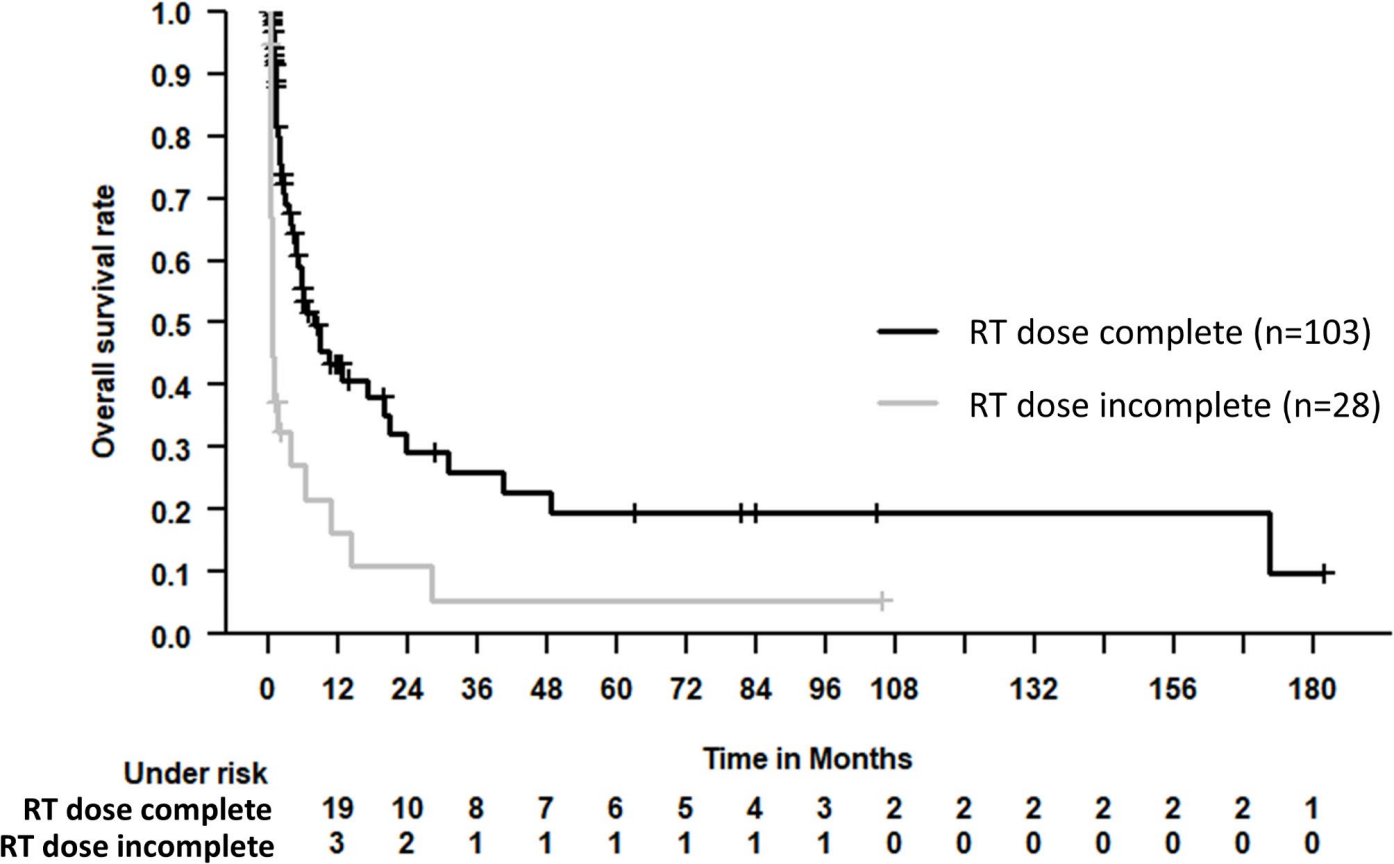
Kaplan-Meier estimate of OS, stratified by completion of RT course

**Fig. 4:**
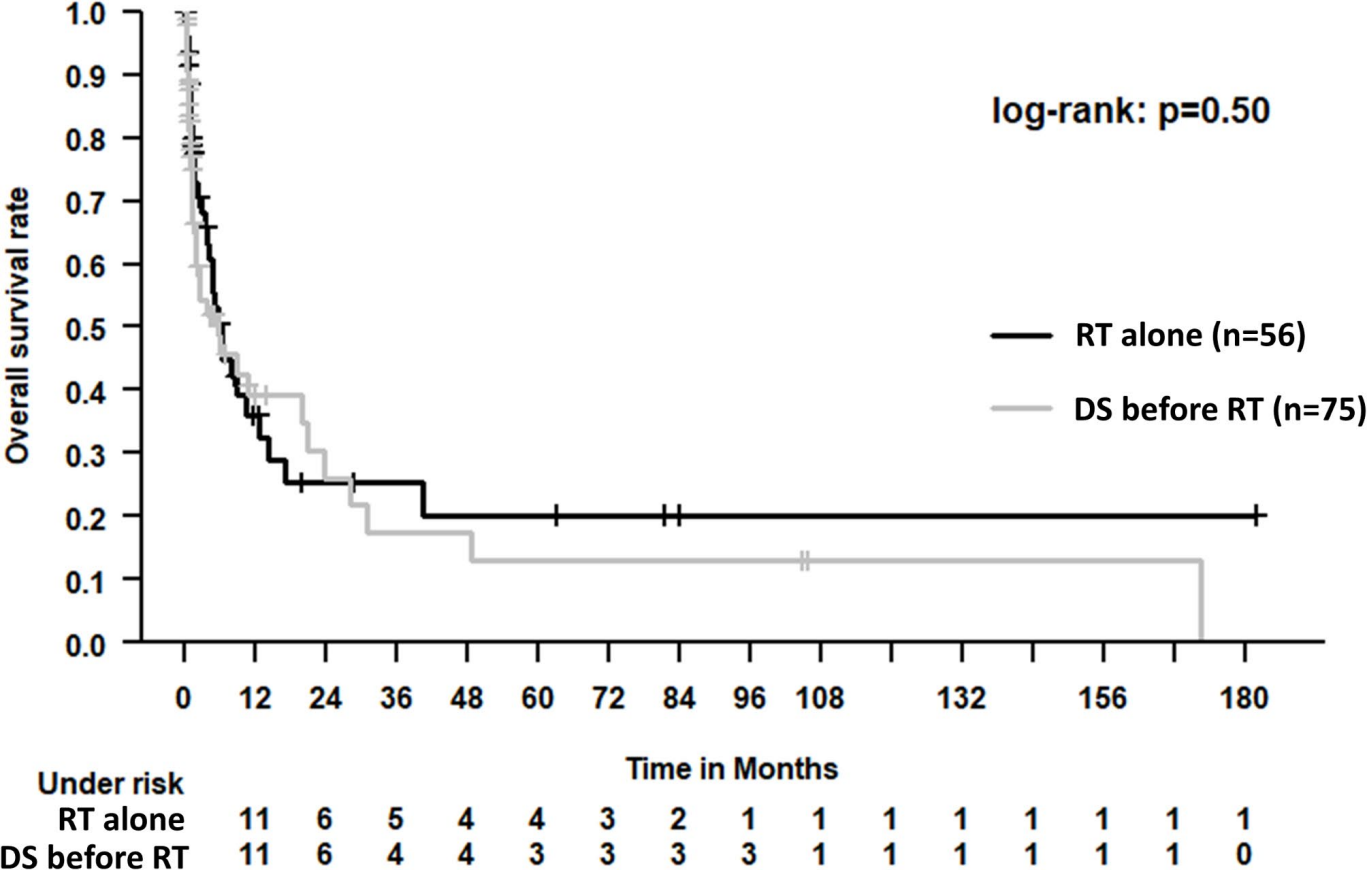
Kaplan-Meier estimate of OS, stratified by surgery

The median OS for the entire patient cohort was 5.6 months (95% confidence interval 3.5–7.7 months). See Fig. 2 for Kaplan-Meier estimates of OS. Figure 3 depicts a Kaplan-Meier estimate of OS, stratified by completion of RT course; Fig. 4 likewise stratified by DS before RT vs. RT alone.

To evaluate factors potentially affecting OS, we first conducted univariable Cox regression. This analysis highlights the strongest most favorable impact on OS for “RT completed as intended” and for “symptom relief achieved”, followed by “BED”. As stated above, there was substantial inter-correlation between “RT completed as intended” with both “RT completed without interruption of ≥ 3 consecutive days” and “BED”. Thus, the two latter were not further considered in the multivariable model. Furthermore, none of the remaining independent variables showed regression coefficients > 0.5 in the correlation matrix of this model. The parameter estimators did not much change between univariable and multivariable analysis. The effect of “Symptom relief achieved” was retained in the multivariable model as it was the “time interval between first and last patient irradiated”. The impact of the parameter “RT completed as intended” was weaker than in the univariable model, albeit still present. Application of any systemic therapy within ± 12 months of radiotherapy was borderline-related with worse OS, possibly due to patients with more advanced tumor disease. Please refer to Table 6 for details.

**Table 6:** Cox regression concerning variables in relation to OS

Variable	Overall survival			
	Univariable		Multivariable	
	Hazard ratio (95%-CI)	*P*-value	Hazard ratio (95%-CI)	*P*-value
Age	1.00 (0.98–1.02)	0.93	1.00 (0.97–1.02)	0.76
Sex female (52) vs. male (79)	0.73 (0.43–1.24)	0.25	0.98 (0.50–1.93)	0.95
CCI > 6 (19) vs. ≤ 6 (112)	1.35 (0.70–2.59)	0.37	1.42 (0.69–2.92)	0.34
SCC as first sign of disease yes (14) vs. no (117)	0.75 (0.37–1.51)	0.42	0.68 (0.29–1.60)	0.38
Relapse of tumor yes (38) vs. no (93)	1.30 (0.79–2.14)	0.30	1.36 (0.73–2.53)	0.33
Spinal metastasis of solid tumor yes (112) vs. no (19)	1.33 (0.69–2.57)	0.40	1.30 (0.59–2.88)	0.52
Systemic therapy^§^ yes (55) vs. no (71)	1.51 (0.93–2.44)	0.09	1.74 (1.00-3.02)	0.05
Surgery conducted prior to radiotherapy, yes (56) vs. no (75)	0.81 (0.50–1.30)	0.38	0.67 (0.39–1.15)	0.15
RT completed as intended yes (103) vs. no (28)	0.33 (0.20–0.55)	2*10 ^**− 5**^	0.48 (0.26–0.86)	**0.01**
RT completed without interruption of ≥ 3 consecutive days, yes (86) vs. no (45)^%^	0.43 (0.26–0.69)	**0.001**		
Time interval between first (01/98) and last (11/18) patient irradiated	1.05 (1.01–1.10)	**0.01**	1.08 (1.03–1.13)	**0.002**
BED [Gy]^%^	0.96 (0.94–0.98)	3*10 ^**− 5**^		
Symptom relief achieved yes (54) no (77)	0.32 (0.19–0.54)	2*10 ^**− 5**^	0.35 (0.19–0.64)	6*10 ^**− 4**^

n.s. = not significant, CCI = Charlson Comorbidity Index, Gy = Gray, SCC = spinal cord compression, RT = Radiotherapy, DS = decompressing surgery. Statistically significant *p*-values are depicted in bold. ^§^Any kind of systemic therapy within 12 months to radiotherapy of spinal manifestations due to SCC. In five cases, dates of systemic therapy were not available and thus were omitted from analysis here. ^%^These two parameters were highly correlated with “RT completed as intended” and were thus not considered for the multivariable linear regression model

### Subgroup analyses

The intent of this study was to address the effects of irradiation on clinical outcome of SCC due to tumor, regardless of the primary malignancy. This involved various tumor entities with potentially different degrees of radiosensitivity. Since the number of cases for individual tumor types was rather small, subgroup analyses were limited. Therefore, we performed such analyses only for the two most frequent tumor entities in our cohort, i.e. breast (*n* = 27) and prostate (*n* = 24) cancer as well as for tumors grouped into three different radiosensitivity grades. These analyses were performed for both endpoints: symptom relief and OS. We restricted these analyses to univariable calculations as multivariable models seem inappropriate with these sample numbers. Nevertheless, some interesting findings were observed at a nominally statistical level of *p* < 0.05.

In breast cancer, older age was associated with a lower probability of achieving symptom relief, whereas prior DS and increasing biologically effective dose (BED) were linked to a higher likelihood of this outcome (Supplemental Table 1). Even stronger effects were observed in relation to OS. “Completion of RT as intended” substantially reduced the HR for OS to one-sixth, with a reasonable confidence interval despite the low sample size. This parameter showed high collinearity with the two variables “RT completed without interruption of ≥ 3 consecutive days” and “BED”, as already seen in the overall cohort (Table 6). Furthermore, achieving symptom relief emerged as a strong predictor for improved OS. In contrast, a CCI > 6 negatively impacted OS, as did RT administered further in the past. Unlike the findings in breast cancer, no associations at *p* < 0.05 were noted with respect to symptom relief or OS in the prostate cancer subgroup (Supplemental Table 2).

Tumor entities were grouped according to low, intermediate, and high radiosensitivity (see Table 2). In tumors with low radiosensitivity (*n* = 22), no associations at *p* < 0.05 were observed between the investigated variables and symptom relief (Supplemental Table 3). However, “completion of radiotherapy as intended” and “BED”, which were closely correlated with one another, came along with markedly improved OS. Completion of RT was linked to a considerably reduced HR to 0.11 (95%-CI 0.02–0.55, *p* = 0.007). Despite the relatively small sample size, the upper limit of the 95%-CI remained well below 1.0.

For tumors with intermediate radiosensitivity, “RT completed as intended” and the closely linked variables “RT completed without interruption” and “BED” predicted an increased probability of symptom relief (Supplemental Table 4). However, this did not translate into improved OS. Instead, OS was predicted by sex (favoring females), CCI (higher index associated with worse outcome), time interval since the first patient was included (worse for earlier years), and if symptom relief was achieved (favorable).

In the high radiosensitivity group, none of the investigated variables predicted symptom relief (Supplemental Table 5). However, OS was impacted by three interrelated variables with “RT completed as intended” demonstrating a remarkably beneficial hazard ratio of 0.14 (95%-CI 0.04–0.54, *p* = 0.004). Symptom relief elicited as a further variable with a relatively strong favorable effect on OS in this context.

An objective of this study was to determine whether patients benefit from surgery prior to RT in terms of symptom relief. Across the entire study population, no such association was found, with an odds ratio of 1.66 (95%-CI 0.82–3.35, *p* = 0.16, Table 5). However, subgroup analyses revealed odds ratios greater than 1.0 for patients with intermediate (Supplemental Table 4) and high (Supplemental Table 5) radiosensitivity, but not for those with low radiosensitivity (Supplemental Table 3). This observation prompted us to conduct an analysis on the combined group of patients with intermediate and high radiosensitivity (*n* = 95). In this group, surgery performed prior to RT was associated with a higher likelihood of symptom relief in both univariable (odds ratio 2.32, 95%-CI 1.01–5.34, *p* = 0.049) and multivariable (2.94, 1.00-8.66, *p* = 0.050, adjusted for the same variables as in Table 5) analyses. The strongest predictor in this multivariable model, by far, remained completion of radiotherapy (odds ratio 21.3, 3.05-148.73, *p* = 0.002). Given the sample size of 95, here we consider multivariable analysis to be appropriate in this context. Interestingly, the extent of symptom relief achieved did not differ significantly among the three radiosensitivity groups (i.e., 9/22, 33/73, 8/22 for low, intermediate, and high sensitivity; *p* = 0.75 according to chi-square test).

## Discussion

We herein report treatment and outcome-related data of 131 patients presenting with acute neurological symptoms due to tumorous SCC, who received RT as part of their emergency treatment in between 1998 and 2018.

In our cohort, symptom relief was achieved in 41.2% of the patients, with apparent better outcomes when DS was performed ahead of RT (45.6%) than RT alone (37.8%). Outcome of RT alone in our cohort is in line with retrospective data published in 2007, regarding RT alone in MSCC for oligometastatic disease [15]. Herein, motor function improvement was achieved in 40% (*n* = 207) patients, whereas 54% (*n* = 279) remained stable motor function. In MSCC due to oligometastatic, relative radioresistant tumors (renal cell carcinoma, colorectal cancer, malignant melanoma), neuronal function was improved in 54% of patients receiving 30 Gy, the median dose also used in our cohort, and 40% in dose escalation [16]. Another study reports about 40% of symptom remission in a cohort of patients with relatively favorable prognosis [17]. In contrast, in a prospective cohort of ten patients with MSCC due to NSCLC, emergency RT was inefficient, generating symptom relief in only two patients [18].

Concerning the impact of surgery, recent retrospective publications report an improvement of neurological functions following laminectomy in about 60% of patients (*n* = 62). Primary tumors were prostate (40%), lung (23%) and breast cancer (11%), similar to our cohort [19].

Discussing the effects of RT alone or DS followed by RT, it is of major importance to take results of the prospective trial performed by Patchell et al. into account [8] (*n* = 101). Herein, DS before RT was compared to RT alone (30 Gy in 10 fractions in both treatment arms), resulting in statistically significant better post-treatment ambulatory rates, significantly longer gait functions and significantly better OS (median 4.2 months when DS followed by RT, 3.3 month when RT alone performed). This trial led to an increasing number of operations for MSCC. Nevertheless, due to a number of severe limitations, the results of this single prospective trial are under an ongoing discussion in the current literature [1, 11, 20, 21]. A matched-pair study published in 2010 did not reproduce these findings, reporting no statistically significant differences when DS with RT was compared to RT alone. For this study, patients suffering from certain conditions (namely, bony fragments in the spinal canal and vertebral fractures), wherein RT alone can hardly be sufficient, were purposely excluded.

Most recently, Rades et al. performed a high-quality matched-pair study comparing patients treated by DS followed by RT to patients receiving RT alone [11]. Herein, in line with Patchell et al., improvement of motor function occurred more often (*p* = 0.015) when performing DS followed by RT in comparison to RT alone. Notably, more than a third of DS patients did not finish RT because of worsening general condition or early death. This was not the case in our study (RT alone: 78.4% completed as intended, DS before RT: 82.1%). However, median OS was 5.6 months with no statistically significant difference between patient groups. This appears consistent compared with previously published studies specifically for patients with short life expectancy: In a phase III trial evaluating two different hypofractionated RT schedules, median OS was 3 months in those being able to walk before MSCC and 2 months for nonwalking patients [22]; median OS in the SCORAD III trial was above 3 months [23].

Bearing in mind that patients suffering from MSCC show generally relative short survival times, it is desirable to find factors helping to determine which patients exactly benefit from surgery. Aiming at the estimated survival times, several clinical and preclinical factors have been identified [24, 25], helping to determine individually tailored therapies in a multiprofessional emergency situation.

Indication for DS besides spinal stability and neurologic deficits should be weighted carefully in terms of benefits and potential harm, as surgery-associated complications have been shown to occur frequently within this patient group (26–29% in retrospective analysis; [26, 27]), and younger patients (< 65 years) were demonstrated to benefit more from surgical interventions [28]. RT dose fractionation has to be individualized and specifically tailored to general condition and oncological status as well, as several studies show no differences in neurological status or postambulatory rates when using short-term RT courses for those with an expected low survival time (e.g., < 6 months) [23, 29, 30]. Furthermore, recent retrospective data support the use of stereotactic body RT (SBRT), which appears to be a valuable approach for surgically unfit patients: Patel et al. report 92.5% local control 1 year after treatment and maintained or improved ambulatory status of 67% [31]. Nevertheless, a recent analysis of practice patterns on SBRT for metastatic spine from lower- and middle-income countries indicated that a vast majority of patients worldwide do not have access to these highly sophisticated treatment options [32].

Rades et at. suggest short-course RT for patients with poor prognosis (e.g., 1*8 Gy), 5*5 Gy for intermediate prognosis patients and longer-course programs (e.g., 10*3 Gy up to 20*2 Gy) for patients with good prognosis [33]. Furthermore, Rades et al. have also suggested an approach to identify patients with an expected survival time of ≤ 2 months, who appear to benefit from RT as opposed to best supportive care [34, 35]. This approach has only recently been introduced to our institution and is not yet represented in our analysis: in our cohort, there was no stereotactic body radiotherapy (SBRT), no single fractions above 3 Gy, no simultan integrated boost concepts.

Interpreting our data, there are further limitations to consider. Primarily, all data was gathered in a retrospective setting, therefore, several bias have to be taken into account. We chose any improvement of clinically determined improvement of neurological function as primary endpoint, which we were not able to detail any further due to inconsistent and/or lacking scoring. The same accounts for widely used prognostic scores (such as the revised versions of the Tokuhashi score, the Dutch Bone Metastasis-Study-Score, the Rades-Score or the Hoskin-Nomogram), which have not been used consistently in our patient cohort [36–40]. Therefore, we cannot give details to the extent of achieved improvement, which has to be assessed as a major shortcoming of the data. Furthermore, the highly important timeframe from the onset of neurological symptoms to RT initiation or DS could not be determined in terms of hours. However, all patients received emergency treatment within 24 h after first emergency ward presentation.

Discussing quality of life metrics, due to the retrospective nature of our data, we are not able to report and interpret Quality of Life (QoL) data from our cohort beyond that defined as primary endpoint in the Methods section, i.e. symptom relief in terms of any clinically determined improvement of neurological functions. QoL has emerged as a major outcome parameter especially in palliative treatment regimens [41]. A recent review by the EORTC Quality of Life Group highlights different symptom categories for patients suffering from MSCC: direct symptoms, such as back pain, paralysis, limb weakness and incontinence; indirect, treatment related symptoms like dysphagia, diarrhea, fatigue and psychosocial concerns like depression and fear about their diagnosis and future. These aspects need to be addressed in a prospective fashion [42].

Radiosensitivity of different malignancies varies widely. We have taken account of this aspect in subgroup analysis. First, in the two largest patient groups (primary tumor breast cancer and prostate cancer): whereas in prostate cancer patients, no statistical significant relation was noted, breast cancer patients were found to have a substantially reduced HR for OS when RT was completed as intended (Supplemental Table 1). Achieving symptom relief was higher when DS was performed and more likely by increasing BED. Analyzing subgroups of different radiosensitivity, we found that patients with tumors of intermediate or high radiosensitivity may benefit from DS prior to RT, in contrast to those with malignancies of low radiosensitivity. At first glance, this may seem counterintuitive, as one might expect DS to be particularly relevant in cases of low radiosensitivity. However, the small sample size of this group (*n* = 22) limits the strength of any conclusions at this point. Nevertheless, the borderline association between prior DS and symptom relief in intermediate or high radiosensitive tumors– based on a more substantial sample of 95 cases– suggests that surgery might provide some benefit for symptom relief. However, even in this group, the effect of DS was much lower than that of completing RT as planned.

Retrospective data concerning the outcome of RT on different radiosensitive tumors in bone metastasis exist for conventional fractionation as well as specifically for the outcome of spinal metastasis by SBRT [43–45]. Whereas local control of bone metastasis was reported as worse for radioresistant tumors in conventionally or moderately hypofractionated RT, radiosensitivity did not impact the clinical outcome when SBRT was administered. In our data, these findings have to be interpreted with high caution: small sample sizes have to be taken into account, as well as the fact that patients not achieving the fully administered dose were likely in a worse general condition, therefore possibly explaining the worse OS.

Median OS in our cohort was 5.6 months, making the primary endpoint of symptom relief difficult to interpret. Inclusion of 21 years of practice covers different preferred procedures, tending towards RT alone in the earlier years and towards DS followed by RT in years that are more recent. Multivariably tested, the timeframe did show a statistically significant impact on OS in favor of more recent treatment dates. This might represent a variety of technical advances in diagnostic imaging, RT treatment planning and delivery as well as changes in peri- and intraoperative care. Furthermore, quickly emerging systemic treatments such as targeted therapies and immunotherapies may have a profound effect on OS of metastasized patients with or without combining RT [46].

Keeping those limitations in mind, we present a rather large study cohort (*n* = 131), covering treatment decisions and outcome of 21 years in a wide variety of malignant spinal cord compressions. These data represent the entire spectrum covered in an academic tumor center, implying locoregional state-of-the-art interdisciplinary management and treatment strategies at the current treatment date. Despite the short median OS, our extended follow up was able to demonstrate seven patients surviving five years; two patients were still alive more than 14 years after treatment.

## Conclusion

When considering the entire patient cohort, no statistically significant difference in neurological symptom remission was observed between patients with *versus* without decompressive surgery prior to RT. However, subgroup analyses may suggest a potential advantage of surgery prior to RT. The strongest and most consistent finding related to OS was the achievement of neurological symptom relief, which, in turn, was primarily predicted by the completion of the radiotherapy course as intended.

## Electronic supplementary material

Below is the link to the electronic supplementary material.

Supplementary Material 1

## Data Availability

No datasets were generated or analysed during the current study.

## Abbreviations

CCI: Charlson Comorbidity Index
DS: Decompressive surgery
Gy: Gray
MSCC: Malignant spinal cord compression
OS: Overall survival
QoL: Quality of Life
RT: Radiotherapy
SBRT: Stereotactic body radiotherapy
SCC: Spinal cord compression
VMAT: Volumetric modulated Arc Therapy
3DcRT: 3D conformal Radiotherapy

## References

[1] Lawton AJ, Lee KA, Cheville AL, Ferrone ML, Rades D, Balboni TA, et al. Assessment and management of patients with metastatic spinal cord compression: A multidisciplinary review. J Clin Oncol. 2019;37(1):61–71. 30395488 10.1200/JCO.2018.78.1211

[2] Levack P, Graham J, Collie D, Grant R, Kidd J, Kunkler I, et al. Don’t wait for a sensory level–listen to the symptoms: a prospective audit of the delays in diagnosis of malignant cord compression. Clin Oncol (R Coll Radiol). 2002;14(6):472–80. 12512970 10.1053/clon.2002.0098

[3] Prasad D, Schiff D. Malignant spinal-cord compression. Lancet Oncol. 2005;6(1):15–24. 15629272 10.1016/S1470-2045(04)01709-7

[4] Souchon R, Wenz F, Sedlmayer F, Budach W, Dunst J, Feyer P, et al. DEGRO practice guidelines for palliative radiotherapy of metastatic breast cancer: bone metastases and metastatic spinal cord compression (MSCC). Strahlenther Onkol. 2009;185(7):417–24. 19714302 10.1007/s00066-009-2044-2

[5] Byrne TN. Spinal cord compression from epidural metastases. N Engl J Med. 1992;327(9):614–9. 1296600 10.1056/NEJM199208273270907

[6] Loblaw DA, Perry J, Chambers A, Laperriere NJ. Systematic review of the diagnosis and management of malignant extradural spinal cord compression: the cancer care Ontario practice guidelines initiative’s Neuro-Oncology disease site group. J Clin Oncol. 2005;23(9):2028–37. 15774794 10.1200/JCO.2005.00.067

[7] Fehlings MG, Nater A, Tetreault L, Kopjar B, Arnold P, Dekutoski M, et al. Survival and clinical outcomes in surgically treated patients with metastatic epidural spinal cord compression: results of the prospective multicenter aospine study. J Clin Oncol. 2016;34(3):268–76. 26598751 10.1200/JCO.2015.61.9338

[8] Patchell RA, Tibbs PA, Regine WF, Payne R, Saris S, Kryscio RJ, et al. Direct decompressive surgical resection in the treatment of spinal cord compression caused by metastatic cancer: a randomised trial. Lancet. 2005;366(9486):643–8. 16112300 10.1016/S0140-6736(05)66954-1

[9] Rades D, Huttenlocher S, Dunst J, Bajrovic A, Karstens JH, Rudat V, et al. Matched pair analysis comparing surgery followed by radiotherapy and radiotherapy alone for metastatic spinal cord compression. J Clin Oncol. 2010;28(22):3597–604. 20606090 10.1200/JCO.2010.28.5635

[10] Rades D, Huttenlocher S, Bajrovic A, Karstens JH, Adamietz IA, Kazic N, et al. Surgery followed by radiotherapy versus radiotherapy alone for metastatic spinal cord compression from unfavorable tumors. Int J Radiat Oncol Biol Phys. 2011;81(5):e861–8. 21277114 10.1016/j.ijrobp.2010.11.056

[11] Rades D, Küchler J, Graumüller L, Abusamha A, Schild SE, Gliemroth J. Radiotherapy with or without decompressive surgery for metastatic spinal cord compression: A retrospective Matched-Pair study including data from prospectively evaluated patients. Cancers (Basel) 2022; 14(5). 10.3390/cancers14051260 PMC8909302 35267568

[12] Mattes MD, Nieto JD. Quality improvement initiative to enhance multidisciplinary management of malignant extradural spinal cord compression. JCO Oncol Pract. 2020;16(8):e829–34. 32384016 10.1200/JOP.19.00593 PMC7587429

[13] U.S. DEPARTMENT OF HEALTH AND HUMAN SERVICES. Common Terminology Criteria for Adverse Events (CTCAE) [Version 5.0, November 27, 2017]. 2017. Available from: URL: https://ctep.cancer.gov/protocoldevelopment/electronic_applications/docs/ctcae_v5_quick_reference_5x7.pdf

[14] Gross A, Ziepert M, Scholz M. KMWin–a convenient tool for graphical presentation of results from Kaplan-Meier survival time analysis. PLoS ONE. 2012;7(6):e38960. 22723912 10.1371/journal.pone.0038960 PMC3376120

[15] Rades D, Veninga T, Stalpers LJA, Basic H, Rudat V, Karstens JH, et al. Outcome after radiotherapy alone for metastatic spinal cord compression in patients with oligometastases. J Clin Oncol. 2007;25(1):50–6. 17194905 10.1200/JCO.2006.08.7155

[16] Freundt K, Meyners T, Bajrovic A, Basic H, Karstens JH, Adamietz IA, et al. Radiotherapy for oligometastatic disease in patients with spinal cord compression (MSCC) from relatively radioresistant tumors. Strahlenther Onkol. 2010;186(4):218–23. 20354660 10.1007/s00066-010-2110-9

[17] Rades D, Panzner A, Rudat V, Karstens JH, Schild SE. Dose escalation of radiotherapy for metastatic spinal cord compression (MSCC) in patients with relatively favorable survival prognosis. Strahlenther Onkol. 2011;187(11):729–35. 22037654 10.1007/s00066-011-2266-y

[18] Rief H, Heinhold RC, Petersen LC, Rieken S, Bruckner T, Moghaddam-Alvandi A, et al. Neurological outcome after emergency radiotherapy in MSCC of patients with non-small cell lung cancer–a prospective trial. Radiat Oncol. 2013;8:297. 24373638 10.1186/1748-717X-8-297 PMC3904469

[19] Younsi A, Riemann L, Scherer M, Unterberg A, Zweckberger K. Impact of decompressive laminectomy on the functional outcome of patients with metastatic spinal cord compression and neurological impairment. Clin Exp Metastasis. 2020;37(2):377–90. 31960230 10.1007/s10585-019-10016-z PMC7138774

[20] Knisely J, Strugar J. Can decompressive surgery improve outcome in patients with metastatic epidural spinal-cord compression? Nat Clin Pract Oncol. 2006;3(1):14–5. 16407872 10.1038/ncponc0399

[21] n den Bent MJ. Surgical resection improves outcome in metastatic epidural spinal cord compression. Lancet. 2005;366(9486):609–10. 16112282 10.1016/S0140-6736(05)66955-3

[22] Maranzano E, Bellavita R, Rossi R, de Angelis V, Frattegiani A, Bagnoli R, et al. Short-course versus split-course radiotherapy in metastatic spinal cord compression: results of a phase III, randomized, multicenter trial. J Clin Oncol. 2005;23(15):3358–65. 15738534 10.1200/JCO.2005.08.193

[23] Hoskin PJ, Hopkins K, Misra V, Holt T, McMenemin R, Dubois D, et al. Effect of Single-Fraction vs multifraction radiotherapy on ambulatory status among patients with spinal Canal compression from metastatic cancer: the SCORAD randomized clinical trial. JAMA. 2019;322(21):2084–94. 31794625 10.1001/jama.2019.17913 PMC6902166

[24] Rades D, Fehlauer F, Schulte R, Veninga T, Stalpers LJA, Basic H, et al. Prognostic factors for local control and survival after radiotherapy of metastatic spinal cord compression. J Clin Oncol. 2006;24(21):3388–93. 16849752 10.1200/JCO.2005.05.0542

[25] Rades D, Cacicedo J, Lomidze D, Al-Salool A, Segedin B, Groselj B et al. Prognostic value of preclinical markers after radiotherapy of metastatic spinal cord Compression-An additional analysis of patients from two prospective trials. Cancers (Basel) 2022; 14(10). 10.3390/cancers14102547 PMC9139528 35626151

[26] Iida K, Matsumoto Y, Setsu N, Harimaya K, Kawaguchi K, Hayashida M, et al. The neurological outcome of radiotherapy versus surgery in patients with metastatic spinal cord compression presenting with myelopathy. Arch Orthop Trauma Surg. 2018;138(1):7–12. 29030689 10.1007/s00402-017-2817-5 PMC5754404

[27] Kim JM, Losina E, Bono CM, Schoenfeld AJ, Collins JE, Katz JN, et al. Clinical outcome of metastatic spinal cord compression treated with surgical excision ± radiation versus radiation therapy alone: a systematic review of literature. Spine (Phila Pa 1976). 2012;37(1):78–84. 21629164 10.1097/BRS.0b013e318223b9b6 PMC3876411

[28] Chi JH, Gokaslan Z, McCormick P, Tibbs PA, Kryscio RJ, Patchell RA. Selecting treatment for patients with malignant epidural spinal cord compression-does age matter? Results from a randomized clinical trial. Spine (Phila Pa 1976). 2009;34(5):431–5. 19212272 10.1097/BRS.0b013e318193a25b

[29] Rades D, Stalpers LJA, Veninga T, Schulte R, Hoskin PJ, Obralic N, et al. Evaluation of five radiation schedules and prognostic factors for metastatic spinal cord compression. JCO. 2005;23(15):3366–75. 10.1200/JCO.2005.04.754 15908648

[30] Rades D, Šegedin B, Conde-Moreno AJ, Garcia R, Perpar A, Metz M, et al. Radiotherapy with 4 Gy × 5 versus 3 Gy × 10 for metastatic epidural spinal cord compression: final results of the SCORE-2 trial (ARO 2009/01). J Clin Oncol. 2016;34(6):597–602. 26729431 10.1200/JCO.2015.64.0862

[31] Patel PP, Cao Y, Chen X, LeCompte MC, Kleinberg L, Khan M, et al. Oncologic and functional outcomes after stereotactic body radiation therapy for High-Grade malignant spinal cord compression. Adv Radiat Oncol. 2024;9(1):101327. 38260225 10.1016/j.adro.2023.101327 PMC10801652

[32] Pathak RS, Tibdewal A, Kinhikar R, Wakeham K, Akbarov K, Cordero L, et al. Practice patterns and perspectives on stereotactic body radiation therapy for the metastatic spine from Lower- and Middle-Income countries. JCO Glob Oncol. 2022;8:e2200167. 36103640 10.1200/GO.22.00167 PMC9812512

[33] Rades D, Schild SE. Personalization of radiation therapy in the primary treatment of malignant epidural spinal cord compression (MESCC). Semin Radiat Oncol. 2023;33(2):148–58. 36990632 10.1016/j.semradonc.2022.11.005

[34] Rades D, Segedin B, Schild SE, Lomidze D, Veninga T, Cacicedo J. Identifying patients with malignant spinal cord compression (MSCC) near end of life who can benefit from palliative radiotherapy. Radiat Oncol. 2022;17(1):143. 35978340 10.1186/s13014-022-02117-z PMC9387005

[35] Kerstens P, Yi M, James M. Radiotherapy for metastatic spinal cord compression; can the Rades score predict survival? Asia Pac J Clin Oncol. 2019;15(6):331–6. 31436904 10.1111/ajco.13232

[36] Hoskin PJ, Hopkins K, Misra V, Holt T, McMenemin R, McKinna F, et al. Prognostic factors for survival and ambulatory status at 8 weeks with metastatic spinal cord compression in the SCORAD randomised trial. Radiother Oncol. 2022;173:77–83. 35618101 10.1016/j.radonc.2022.05.017

[37] Rades D, Cacicedo J, Lomidze D, Al-Salool A, Segedin B, Groselj B, et al. A new and Easy-to-Use survival score for patients irradiated for metastatic epidural spinal cord compression. Pract Radiat Oncol. 2022;12(4):354–62. 35395423 10.1016/j.prro.2022.03.012

[38] n der Linden YM, Dijkstra SPDS, Vonk EJA, Marijnen CAM, Leer JWH. Prediction of survival in patients with metastases in the spinal column: results based on a randomized trial of radiotherapy. Cancer. 2005;103(2):320–8. 15593360 10.1002/cncr.20756

[39] Morgen SS, Fruergaard S, Gehrchen M, Bjørck S, Engelholm SA, Dahl B. A revision of the Tokuhashi revised score improves the prognostic ability in patients with metastatic spinal cord compression. J Cancer Res Clin Oncol. 2018;144(1):33–8. 28986702 10.1007/s00432-017-2519-y PMC11813445

[40] Tokuhashi Y, Uei H, Oshima M, Ajiro Y. Scoring system for prediction of metastatic spine tumor prognosis. World J Orthop. 2014;5(3):262–71. 25035829 10.5312/wjo.v5.i3.262 PMC4095019

[41] Kaasa S, Loge JH. Quality-of-life assessment in palliative care. Lancet Oncol. 2002;3(3):175–82. 11902505 10.1016/s1470-2045(02)00682-4

[42] Gojsevic M, Shariati S, Chan AW, Bonomo P, Zhang E, Kennedy SKF, et al. Quality of life in patients with malignant spinal cord compression: a systematic review. Support Care Cancer. 2023;31(12):736. 38055061 10.1007/s00520-023-08186-4

[43] Guo L, Xu Q, Ke L, Wu Z, Zeng Z, Chen L, et al. The impact of radiosensitivity on clinical outcomes of spinal metastases treated with stereotactic body radiotherapy. Cancer Med. 2023;12(12):13279–89. 37162297 10.1002/cam4.6019 PMC10315727

[44] Makita K, Hamamoto Y, Kanzaki H, Kataoka M, Yamamoto S, Nagasaki K, et al. Local control of bone metastases treated with external beam radiotherapy in recent years: a multicenter retrospective study. Radiat Oncol. 2021;16(1):225. 34801042 10.1186/s13014-021-01940-0 PMC8605549

[45] Makita K, Hamamoto Y, Kanzaki H, Nagasaki K, Takata N, Tsuruoka S, et al. Factors affecting local control of bone metastases from radioresistant tumors treated with palliative external beam radiotherapy. Discov Oncol. 2023;14(1):74. 37212949 10.1007/s12672-023-00651-0 PMC10203063

[46] Pitroda SP, Chmura SJ, Weichselbaum RR. Integration of radiotherapy and immunotherapy for treatment of oligometastases. Lancet Oncol. 2019;20(8):e434–42. 31364595 10.1016/S1470-2045(19)30157-3

