# Supplementary material for: Primary and postoperative radiotherapy in acute neurological symptoms due to malignant spinal compression: retrospective analysis from a German university hospital
Source: BMC Cancer. 2025 Apr 23;25:759. doi: 10.1186/s12885-025-14106-y (PMC12016431; doi:10.1186/s12885-025-14106-y)
Supplement: Supplementary file 1 — Supplementary Material 1 [file 12885_2025_14106_MOESM1_ESM.docx]

Supplemental Table 1. Subgroup of patients with breast cancer primary (n=27), univariable analyses in regard to symptom relief (assessed by binary logistic regression) and overall survival (Cox proportional hazard regression)

| **Variable (n)** | **Symptom relief** | | **Overall survival** | |
| --- | --- | --- | --- | --- |
|  | Odds ratio (95%-CI) | P-value | Hazard ratio (95%-CI) | P-value |
| Age [per year] | 0.92 (0.85-0.99) | **0.03** | 1.02 (0.98-1.06) | 0.40 |
| Sex  Female (25) vs male (2) | n.a. | n.a. | 0.50 (0.10-2.46) | 0.40 |
| CCI  >6 (4) vs ≤ 6 (23) | n.a. | n.a. | 5.53 (1.10-27.91) | **0.04** |
| SCC as first sign of disease  yes (2) vs no (25) | 1.27 (0.07-22.72) | 0.87 | 1.25 (0.25-6.15) | 0.78 |
| Relapse of tumor  yes (14) vs no (13) | 0.88 (0.19-3.40) | 0.86 | 1.56 (0.45-5.36) | 0.48 |
| Spinal metastasis of solid tumor  yes (26) vs no (1) | n.a. | n.a. | 0.20 (0.02-1.82) | 0.15 |
| Systemic therapy^§^  yes (10) vs no (16) | 2.50 (0.50-12.64) | 0.27 | 1.00 (0.30-3.32) | 0.99 |
| Surgery conducted prior to radiotherapy, yes (12) vs no (15) | 5.50 (1.05-28.88) | **0.04** | 0.38 (0.10-1.38) | 0.14 |
| RT completed as intended  yes (21) vs no (6) | n.a. | n.a. | 0.16 (0.05-0.58) | **0.005** |
| RT completed without interruption of ≥ 3 consecutive days,  yes (18) vs no (9)^%^ | 4.38 (0.71-27.16) | 0.11 | 0.22 (0.06-0.81) | **0.02** |
| Time interval between first (01/98) and last (11/18) patient irradiated  [per year] | 0.97 (0.82-1.14) | 0.68 | 1.12 (1.01-1.25) | **0.03** |
| BED [per Gy]^%^ | 1.10 (1.00-1.21) | **0.05** | 0.94 (0.89-0.99) | **0.02** |
| Symptom relief | - | **-** | 0.14 (0.03-0.67) | **0.01** |

^§^Any kind of systemic therapy within 12 months to radiotherapy of spinal manifestations due to SCC. In one case, date of systemic therapy was not available and thus was omitted from analysis here. ^%^These two parameters were highly correlated with “RT completed as intended”. CCI = Charlson comorbidity index. SCC = spinal cord compression. RT = radiotherapy. BED = Biologically effective dose of radiotherapy. N.a.=not applicable.

Supplemental Table 2. Subgroup of patients with prostate cancer primary (n=24), univariable analyses in regard to symptom relief (assessed by binary logistic regression) and overall survival (Cox proportional hazard regression).

| **Variable (n)** | **Symptom relief** | | **Overall survival** | |
| --- | --- | --- | --- | --- |
|  | Odds ratio (95%-CI) | P-value | Hazard ratio (95%-CI) | P-value |
| Age [per year] | 0.96 (0.86-1.06) | 0.41 | 1.01 (0.95-1.08) | 0.75 |
| Sex  female (0) vs male (24) | n.a. | n.a. | n.a. | n.a. |
| CCI  >6 (2) vs ≤ 6 (22) | n.a. | n.a. | 4.48 (0.82-24.65) | 0.09 |
| SCC as first sign of disease  yes (1) vs no (23) | n.a. | n.a. | 2.03 (0.24-17.34) | 0.52 |
| Relapse of tumor  yes (8) vs no (16) | 0.33 (0.05-2.18) | 0.25 | 1.15 (0.34-3.85) | 0.82 |
| Spinal metastasis of solid tumor  yes (24) vs no (0) | n.a. | n.a. | n.a. | n.a. |
| Systemic therapy^§^  yes (3) vs no (20) | 3.00 (0.23-38.88) | 0.40 | 1.03 (0.21-5.07) | 0.98 |
| Surgery conducted prior to radiotherapy, yes (13) vs no (11) | 3.11 (0.56-17.33) | 0.20 | 0.83 (0.20-3.42) | 0.80 |
| RT completed as intended  yes (21) vs no (3) | n.a. | n.a. | 0.45 (0.09-2.27) | 0.34 |
| RT completed without interruption of ≥ 3 consecutive days,  yes (19) vs no (5)^%^ | 3.60 (0.34-38.48) | 0.29 | 0.43 (0.10-1.83) | 0.26 |
| Time interval between first (01/98) and last (11/18) patient irradiated | 1.02 (0.89-1.17) | 0.74 | 1.11 (0.98-1.25) | 0.11 |
| BED [Gy]^%^ | 1.20 (0.93-1.54) | 0.16 | 1.00 (0.92-1.09) | 0.98 |
| Symptom relief | - | - | 0.77 (0.23-2.58) | 0.67 |

^§^Any kind of systemic therapy within 12 months to radiotherapy of spinal manifestations due to SCC. In one cases, date of systemic therapy was not available and thus was omitted from analysis here. ^%^These two parameters were highly correlated with “RT completed as intended”. CCI = Charlson comorbidity index. SCC = spinal cord compression. RT = radiotherapy. BED = Biologically effective dose of radiotherapy. N.a.=not applicable.

Supplemental Table 3. Subgroup of patients for tumors with low radiosensitivity (n=22), univariable analyses in regard to symptom relief (assessed by binary logistic regression) and overall survival (Cox proportional hazard regression).

| **Variable (n)** | **Symptom relief** | | **Overall survival** | |
| --- | --- | --- | --- | --- |
|  | Odds ratio (95%-CI) | P-value | Hazard ratio (95%-CI) | P-value |
| Age [per year] | 0.98 (0.90-1.06) | 0.55 | 0.98 (0.93-1.03) | 0.48 |
| Sex  Female (9) vs male (13) | 2.81 (0.48-16.43) | 0.25 | 1.23 (0.39-3.92) | 0.72 |
| CCI  >6 (3) vs ≤ 6 (19) | 0.69 (0.05-8.96) | 0.78 | 0.63 (0.08-5.08) | 0.67 |
| SCC as first sign of disease  yes (3) vs no (19) | 0.69 (0.05-8.96) | 0.78 | 0.87 (0.18-4.26) | 0.87 |
| Relapse of tumor  yes (4) vs no (18) | 0.42 (0.04-4.81) | 0.48 | 1.76 (0.45-6.83) | 0.41 |
| Spinal metastasis of solid tumor  yes (20) vs no (2) | 0.67 (0.04-12.27) | 0.79 | 0.81 (0.16-4.22) | 0.80 |
| Systemic therapy^§^  yes (10) vs no (11) | 0.80 (0.14-4.53) | 0.80 | 1.47 (0.41-5.26) | 0.56 |
| Surgery conducted prior to radiotherapy, yes (12) vs no (15) | 0.50 (0.09-2.81) | 0.43 | 1.90 (0.49-7.45) | 0.36 |
| RT completed as intended  yes (18) vs no (4) | n.a. | n.a. | 0.11 (0.02-0.55) | **0.007** |
| RT completed without interruption of ≥ 3 consecutive days,  yes (11) vs no (11)^%^ | 0.69 (0.12-3.78) | 0.67 | 0.55 (0.17-1.75) | 0.31 |
| Time interval between first (01/98) and last (11/18) patient irradiated  [per year] | 1.08 (0.91-1.28) | 0.39 | 1.04 (0.93-1.16) | 0.54 |
| BED [per Gy]^%^ | 1.14 (0.98-1.32) | 0.09 | 0.90 (0.83-0.97) | **0.006** |
| Symptom relief, yes (9) vs (13) | - | **-** | 0.31 (0.08-1.15) | 0.08 |

^§^Any kind of systemic therapy within 12 months to radiotherapy of spinal manifestations due to SCC. In one case, date of systemic therapy was not available and thus was omitted from analysis here. ^%^These two parameters were highly correlated with “RT completed as intended” with respect to OS. CCI = Charlson comorbidity index. SCC = spinal cord compression. RT = radiotherapy. BED = Biologically effective dose of radiotherapy. N.a.=not applicable.

Supplemental Table 4. Subgroup of patients for tumors with intermediate radiosensitivity (n=73), univariable analyses in regard to symptom relief (assessed by binary logistic regression) and overall survival (Cox proportional hazard regression).

| **Variable (n)** | **Symptom relief** | | **Overall survival** | |
| --- | --- | --- | --- | --- |
|  | Odds ratio (95%-CI) | P-value | Hazard ratio (95%-CI) | P-value |
| Age [per year] | 0.96 (0.93-1.00) | 0.05 | 1.02 (0.99-1.04) | 0.17 |
| Sex  Female (35) vs male (38) | 1.04 (0.41-2.62) | 0.93 | 0.48 (0.24-0.97) | **0.04** |
| CCI  >6 (9) vs ≤ 6 (64) | 0.30 (0.06-1.58) | 0.16 | 3.57 (1.49-8.57) | **0.004** |
| SCC as first sign of disease  yes (8) vs no (65) | 2.20 (0.49-10.00) | 0.31 | 0.64 (0.25-1.66) | 0.36 |
| Relapse of tumor  yes (27) vs no (46) | 0.95 (0.37-2.48) | 0.92 | 1.16 (0.60-2.26) | 0.66 |
| Spinal metastasis of solid tumor  yes (70) vs no (3) | 0.40 (0.03-4.59) | 0.46 | 2.39 (0.32-17.82) | 0.40 |
| Systemic therapy^§^  yes (28) vs no (42) | 2.51 (0.94-6.70) | 0.07 | 1.22 (0.63-2.39) | 0.56 |
| Surgery conducted prior to radiotherapy, yes (33) vs no (40) | 2.52 (0.98-6.50) | 0.06 | 0.72 (0.37-1.39) | 0.32 |
| RT completed as intended  yes (59) vs no (14) | 6.64 (1.37-32.31) | **0.02** | 0.56 (0.27-1.17) | 0.12 |
| RT completed without interruption of ≥ 3 consecutive days,  yes (51) vs no (22)^%^ | 3.00 (1.01-8.90) | **0.048** | 0.63 (0.32-1.27) | 0.20 |
| Time interval between first (01/98) and last (11/18) patient irradiated  [per year] | 1.02 (0.94-1.11) | 0.61 | 1.08 (1.02-1.14) | **0.01** |
| BED [per Gy]^%^ | 1.08 (1.01-1.14) | **0.02** | 0.99 (0.95-1.02) | 0.35 |
| Symptom relief, yes (33) vs no (40) | - | **-** | 0.40 (0.20-0.79) | **0.009** |

^§^Any kind of systemic therapy within 12 months to radiotherapy of spinal manifestations due to SCC. In three cases, date of systemic therapy was not available and thus was omitted from analysis here. ^%^These two parameters were highly correlated with “RT completed as intended”. CCI = Charlson comorbidity index. SCC = spinal cord compression. RT = radiotherapy. BED = Biologically effective dose of radiotherapy. N.a.=not applicable.

Supplemental Table 5. Subgroup of patients for tumors with high radiosensitivity (n=22), univariable analyses in regard to symptom relief (assessed by binary logistic regression) and overall survival (Cox proportional hazard regression).

| **Variable (n)** | **Symptom relief** | | **Overall survival** | |
| --- | --- | --- | --- | --- |
|  | Odds ratio (95%-CI) | P-value | Hazard ratio (95%-CI) | P-value |
| Age [per year] | 1.07 (0.97-1.18) | 0.20 | 0.97 (0.90-1.04) | 0.42 |
| Sex  Female (6) vs male (16) | 0.26 (0.02-2.73) | 0.26 | n.r.a. | n.r.a. |
| CCI  >6 (4) vs ≤ 6 (18) | 0.52 (0.05-6.09) | 0.61 | 0.39 (0.05-3.21) | 0.38 |
| SCC as first sign of disease  yes (1) vs no (21) | n.a. | n.a. | 0.50 (0.06-4.23) | 0.53 |
| Relapse of tumor  yes (6) vs no (16) | 2.20 (0.32-14.98) | 0.42 | 1.95 (0.59-6.45) | 0.27 |
| Spinal metastasis of solid tumor  yes (10) vs no (12) | 0.25 (0.04-1.70) | 0.16 | 1.88 (0.57-6.17) | 0.30 |
| Systemic therapy^§^  yes (12) vs no (9) | 0.27 (0.04-1.70) | 0.16 | 3.45 (0.93-12.78) | 0.06 |
| Surgery conducted prior to radiotherapy, yes (7) vs no (15) | 1.50 (0.24-9.47) | 0.67 | 0.44 (0.12-1.64) | 0.22 |
| RT completed as intended  yes (17) vs no (5) | n.a. | n.a. | 0.14 (0.04-0.54) | **0.004** |
| RT completed without interruption of ≥ 3 consecutive days,  yes (16) vs no (6)^%^ | n.a. | n.a. | 0.14 (0.04-0.58) | **0.006** |
| Time interval between first (01/98) and last (11/18) patient irradiated  [per year] | 0.94 (0.81-1.09) | 0.40 | 1.07 (0.97-1.17) | 0.19 |
| BED [per Gy]^%^ | 1.13 (0.96-1.32) | 0.13 | 0.95 (0.91-0.99) | **0.01** |
| Symptom relief, yes (8) vs no (14) | - | **-** | 0.16 (0.04-0.63) | **0.008** |

^§^Any kind of systemic therapy within 12 months to radiotherapy of spinal manifestations due to SCC. In one case, date of systemic therapy was not available and thus was omitted from analysis here. ^%^These two parameters were highly correlated with “RT completed as intended”. CCI = Charlson comorbidity index. SCC = spinal cord compression. RT = radiotherapy. BED = Biologically effective dose of radiotherapy. N.a. = not applicable. N.r.a. = not reasonably applicable, i.e. extraordinary values for point estimator and confidence intervals.
